# Clustering of critically ill patients using an individualized learning approach enables dose optimization of mobilization in the ICU

**DOI:** 10.1186/s13054-022-04291-8

**Published:** 2023-01-03

**Authors:** Kristina E. Fuest, Bernhard Ulm, Nils Daum, Maximilian Lindholz, Marco Lorenz, Kilian Blobner, Nadine Langer, Carol Hodgson, Margaret Herridge, Manfred Blobner, Stefan J. Schaller

**Affiliations:** 1 Technical University Munich, School of Medicine, Klinikum Rechts der Isar, Department of Anaesthesiology & Intensive Care Medicine, Munich, Germany; 2 Charité—Universitätsmedizin Berlin, corporate member of Freie Universität Berlin and Humboldt-Universität Zu Berlin, Department of Anesthesiology and Operative Intensive Care Medicine (CVK, CCM), Berlin, Germany; 3 Technical University Munich, School of Medicine, Klinikum Rechts der Isar, Department of Orthopedics, Munich, Germany; 4 Acute and Critical Care, Monash University, Melbourne, VIC Australia; 5 Interdepartmental Division of Critical Care Medicine, University of Toronto, University Health Network, Toronto, ON Canada; 6 Faculty of Medicine, Department of Anesthesiology and Intensive Care Medicine, University Hospital Ulm, Ulm, Germany

**Keywords:** Early ambulation, Critical care, Critical illness, Physical therapy modalities, Patient discharge

## Abstract

**Background:**

While early mobilization is commonly implemented in intensive care unit treatment guidelines to improve functional outcome, the characterization of the optimal individual dosage (frequency, level or duration) remains unclear. The aim of this study was to demonstrate that artificial intelligence-based clustering of a large ICU cohort can provide individualized mobilization recommendations that have a positive impact on the likelihood of being discharged home.

**Methods:**

This study is an analysis of a prospective observational database of two interdisciplinary intensive care units in Munich, Germany. Dosage of mobilization is determined by sessions per day, mean duration, early mobilization as well as average and maximum level achieved. A k-means cluster analysis was conducted including collected parameters at ICU admission to generate clinically definable clusters.

**Results:**

Between April 2017 and May 2019, 948 patients were included. Four different clusters were identified, comprising “Young Trauma,” “Severely ill & Frail,” “Old non-frail” and “Middle-aged” patients. Early mobilization (< 72 h) was the most important factor to be discharged home in “Young Trauma” patients (OR_adj_ 10.0 [2.8 to 44.0], *p* < 0.001). In the cluster of “Middle-aged” patients, the likelihood to be discharged home increased with each mobilization level, to a maximum 24-fold increased likelihood for ambulating (OR_adj_ 24.0 [7.4 to 86.1], *p* < 0.001). The likelihood increased significantly when standing or ambulating was achieved in the older, non-frail cluster (OR_adj_ 4.7 [1.2 to 23.2], *p* = 0.035 and OR_adj_ 8.1 [1.8 to 45.8], *p* = 0.010).

**Conclusions:**

An artificial intelligence-based learning approach was able to divide a heterogeneous critical care cohort into four clusters, which differed significantly in their clinical characteristics and in their mobilization parameters. Depending on the cluster, different mobilization strategies supported the likelihood of being discharged home enabling an individualized and resource-optimized mobilization approach.

**Trial Registration**: Clinical Trials NCT03666286, retrospectively registered 04 September 2018.

**Supplementary Information:**

The online version contains supplementary material available at 10.1186/s13054-022-04291-8.

## Introduction

Early mobilization has been suggested as a promising intervention to counteract intensive care unit acquired weakness (ICUAW) by attenuating the muscle wasting associated with critical illness [1]. Previous clinical studies with an early intervention start have shown, early mobilization can be safely initiated in the ICU and might improve the functional capacity, reduce days with mechanical ventilation in the ICU and increase the rate of discharge home [1–5]. However, in the recently published TEAM trial increasing active early mobilization did not result in significantly reduced hospital length of stay or mortality compared to usual ICU mobilization. In addition, the rate of adverse events was increased in the intervention group [6]. This raises the question which patients benefit most from early mobilization and how to determine the appropriate type, timing, intensity, coordination and duration of therapy [7]. Wide variability was found in intervention characteristics, outcome measures and associated metrics, leading to conflicting results regarding the influence of early mobilization dosage on functional status after ICU stay [8, 9]. Commonly, only the maximum level of mobilization achieved is quantified, whereas the duration, intensity and frequency as important parameters of the dosage of mobilization are not [10]. Since the group of intensive care patients is extremely heterogeneous, it is difficult to recommend interventions across all patients. The relationship between severity of illness, age, weight and the presence of functional impairment and comorbidities regarding the implementation of rehabilitation also remains unclear [8, 11–13]. As a result, it is difficult to determine the appropriate timing and dose of intervention to achieve the optimal benefit for the respective patient [9, 14, 15].

An individualized approach to early mobilization which considers the pre-existing functional status, frailty, comorbidities, disease severity and invasiveness of the treatment in the ICU might be meaningful. The aim of this study was to test that it is feasible to divide the diverse group of ICU patients into specific cohorts by clustering and derive specific individualized recommendations for mobilization to increase the probability to be discharged home. We hypothesized that identifiable patient groups benefit differently from different mobilization components.

## Methods & materials

### Study design, setting and participants

This study is an analysis of our prospective observational database of two interdisciplinary intensive care units of the Department of Anaesthesiology and Intensive Care at Klinikum rechts der Isar, School of Medicine, Technical University of Munich, Germany between April 2017 and May 2019. The database is registered at Clinical Trials (NCT03666286, registered 04 September 2018) and approved by the Ethics Committee of the Faculty of Medicine, Technical University of Munich (518/16S). Adults with > 24 h stay in the ICU were included in the database, if consent was obtained either by the patient or legal representative according to German legislation. Patients were included in the analysis, whose mobilization during the intensive care unit stay was fully recorded.

### Outcome variables

The primary outcome was discharge disposition “home.” It was tested against the combination of all adverse discharge dispositions (nursing home, hospice, another hospital or death), considering it as the optimal outcome after critical illness as opposed to institutionalization [16, 17]. Secondary outcome variables are ICU mortality, hospital mortality, ICU length of stay (LOS) and hospital LOS.

### Factors of interest

The factor of interest was mobilization using the surgical ICU optimal mobilization score (SOMS) (representing active and passive mobilization) [18–20] and the ICU Mobility Scale (IMS) (representing only active mobilization) [21–23]. The SOMS describes a patient’s mobilization capacity on a numerical rating scale ranging from 0 (no mobilization) to 4 (ambulation) capturing both active and passive mobilization forms [4]. The IMS captures active forms of mobilization and ranges from 0 “lying in bed” to 10 “walking independently without a gait aid” with an excellent inter-rater reliability if used in critically ill patients [21]. To determine the dosage of mobilization, sessions per day, mean duration per day (in minutes), first day of mobilization, average and maximum level reached during ICU stay and the distribution of the individual levels achieved were recorded. Mobilization was defined as “early” if it occurred within the first 72 h after intensive care admission [24, 25]. Mobilization could be performed by all professionals working in the ICU and not exclusively by physiotherapists.

### Data collection

Data included upon admission were basic demographics, location prior to ICU admission, ICU admission category (sepsis, polytrauma, traumatic brain injury, non-traumatic brain injury, postoperative monitoring, cardiac failure, respiratory failure and “other”) and diagnosis (e.g., sepsis or trauma) and several scores to characterize the cohort: baseline Glasgow Coma Scale (GCS), Clinical Frailty Scale (CFS) [26, 27], Charlson Comorbidity Index [28], Sequential Organ Failure Assessment score (SOFA) [29] as well as standard laboratory and hemodynamic parameters. Functional status was assessed using two relevant sub-domains of the Barthel Index, an ordinal scale comprising ten sub-domains of activities of daily living, which is the most used scale for activities of daily living [30, 31]. The scores of the “mobility” and “transfer” sub-domains of the Barthel (“Mobility-Transfer-Barthel,” MTB) index represent a patient’s functional ability and gait independence with a minimum of 0 points (functionally totally dependent) and a maximum of 30 points (functionally independent) [32]. A premorbid baseline value was obtained representing the functional status two weeks before hospital admission. Upon ICU discharge, data was obtained regarding ICU LOS and mortality, as well as data about ICU-related therapy (e.g., fluid administration, nutrition, dialysis and laboratory parameters). Upon hospital discharge, data on LOS and discharge disposition (prior residence, nursing home, rehabilitation clinic, etc.) as well as mortality was obtained. The compilation of these variables led to a comprehensive characterization of our cohort in terms of the feasibility of mobilization. In addition to patient characteristics (age, Body Mass Index, sex), functional status before the critical illness (frailty, Mobility-Transfer-Barthel, Charlson Comorbidity Index) as well as disease severity (SOFA, APACHE II, Glasgow Coma Scale) and condition at ICU admission were recorded in detail.

### Statistical analysis

#### Clustering

Since the underlying patient collective was a very inhomogeneous cohort, an attempt was made to find groups that were as similar as possible and as different from each other as possible. To achieve this goal, a k-means cluster analysis was conducted using the method k-means from the base R with the following factors: *sex, age, Body Mass Index, Mobility-Transfer-Barthel at hospital admission, department (e.g., neurosurgery, cardiology), admission form (e.g., from home, another hospital, nursing home), Clinical Frailty Scale, Glasgow Coma Scale, APACHE 2 score, SOFA score, Charlson Comorbidity Index, and ICU admission categories (sepsis, polytrauma, traumatic brain injury, non-traumatic brain injury, postoperative monitoring, cardiac failure, respiratory failure and “other”)*. A crucial part of the k-means cluster analysis is the selection of k, which represents the number of groups to be identified by the clustering algorithm. We chose the elbow method to determine the optimal number of clusters. Since the cluster analysis is an unsupervised method, the resulting clusters must be named according to the characteristics of the included expressions [33]. Cluster stability assessment was checked using the average Jaccard index on 1000 bootstrap samples [34]. To delineate the performance of the clustering method, Euclidean distances were calculated and plotted [34]. To visualize clustering results, a Principal Component Analysis plot for k-means clustering using the first two components was plotted.

#### Analysis

In each cluster, the influence of the mobilization parameters on the primary endpoint “discharge home” for all patients including patients who died, was analyzed. The influence of the four mobilization parameters on the primary endpoint was analyzed using Mann–Whitney U tests or Fisher's exact tests. Then, a logistic regression model using only the four mobilization parameters was calculated. For model adjustments, stepwise forward logistic regression models were computed. In these models, all mobilization parameters were mandatory to stay in the model and all variables used for clustering were added in a stepwise forward manner, using Akaike Information Criterion as a means of model performance.

The mobilization parameters were also compared between the clusters using Kruskal–Wallis test for continuous variables and Fishers exact test for categorical variables. Post hoc analysis was conducted using Mann–Whitey U tests and Fishers exact tests with Bonferroni correction.

All continuous variables are presented as median with interquartile range [IQR], all categorical variables with absolute and relative numbers. An alpha of 5% was selected as level of significance. We further performed several sensitivity analyses: First, the primary analysis was repeated in survivors only in all patients and the four clusters. Secondly, the primary analysis was repeated with the 11-point IMS Score. All analyses were performed using R version 4.2.1 (R Foundation for Statistical Computing; Vienna Austria).

#### Power analysis

According to Vittinghoff and McCulloch, a case number of 5–9 patients per variable with the smaller outcome category is required to ensure sufficient power [35]. With the four mobilization parameters included in the analysis, we had to ensure that there were at least 20 patients with the smaller outcome category in the cluster.

#### Missing values

Variables with more than 5% missing values were excluded from the study. For continuous variables, median imputation was performed. For categorical variables, a category for the missingness was added.

## Results

**Fig. 1:**
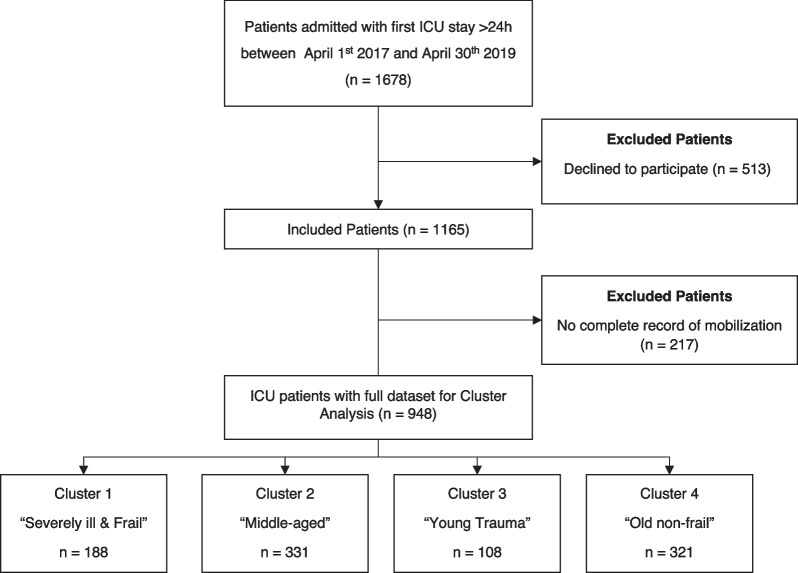
STROBE Diagram

Between April 1st, 2017 and May 31st, 2019, we included 948 patients (Fig. 1). The characteristics of the 300 patients (32%) who could be discharged home differed significantly from all others (see Additional file 1: Table S1).

### Cluster analysis

**Fig. 2:**
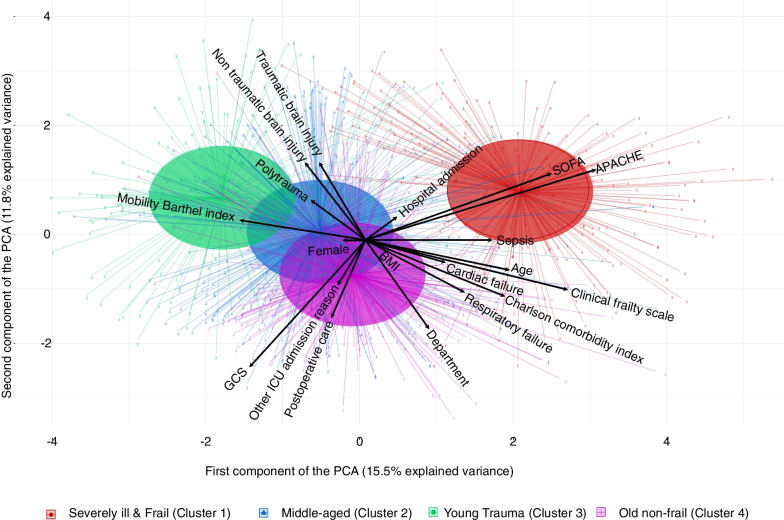
Biplot of the Cluster centers on the first two dimensions of a Principal Components Analysis (PCA). Arrows illustrate the strength and direction of the influence of the variables on the first and the second component of the PCA. The higher the value of a variable, the longer the arrow, and the stronger the influence in the direction of the arrow. The colored ellipses show the cluster centers. The dots in different colors indicate individual patients and their belonging to the clusters. First component of the PCA explains 15.5% of the variance in the data and is highly positively loaded with APACHE, SOFA and frailty scores and highly negatively loaded with Mobility-Transfer-Barthel, GCS, as well as the admission reasons non-traumatic brain injury, tic brain injury and polytrauma. The second component explains 11.8% of the variance in the data and is highly positive loaded with APACHE, non-traumatic brain injury and traumatic brain injury and highly negative loaded with GCS, department and the admission reason postoperative. The red cluster is mainly loaded with high APACHE, SOFA and Clinical Frailty Scale, which is why it is labeled “*Severely ill & Frail*”. The green cluster is mainly loaded with young age, high Mobility-Transfer-Barthel, and polytrauma, which is why it is labeled “*Young Trauma*”. The purple cluster is mainly loaded with allocation for postoperative treatment due to old age but low SOFA, APACHE and Clinical Frailty Scale, which is why it is labeled “*Old non-frail*”. The blue cluster has no specific load from the first or second principal component. Since the cluster’s median age is close to that of the total cohort, it is labeled “*Middle-aged*”. *GCS* Glasgow Coma Scale, *SOFA* Sepsis-related organ failure assessment score, *APACHE* Acute physiology and chronic health evaluation score

No variable had a rate of missing data over 5%. Missing data from BMI, APACHE II and SOFA score had to be imputed with the median. The elbow method showed an optimal number of four clusters (Additional file 1: Figure S1). Each cluster contained clinically determinable patient characteristics and was represented in the Principal Component Analysis plot for k-means clustering (Fig. 2): Clusters were labeled according to the major loads resulting in clusters “*Severely ill & Frail*,” *“Young Trauma,” “Old non-frail”* and *“Middle-aged”*. The patients differed between the clusters in terms of several characteristics (Table 1). Post hoc analyses are presented in Additional file 1: Table S2. Jaccard Index ranged from 0.74 in cluster “*Severely ill & Frail*” patients to 0.94 in cluster “*Young Trauma*”. The Euclidean distance plot can be found in the Additional file 1: Figure S2.

**Table 1:** Patient characteristics in the four clusters

	Severely ill & Frail	Middle-aged	Young Trauma	Old non-frail	*P* value
	(*N* = 188)	(*N* = 331)	(*N* = 108)	(*N* = 321)	
Female sex	107 (56.9)	202 (61.0)	60 (55.6)	185 (57.6)	0.67
**Body Mass Index (kg/m**^**2**^)	25.4 [23.5–27.7]	25.7 [23.1–28.1]	24.2 [21.1–26.7]	25.4 [23.3–28.1]	**0.007**
Underweight	5 (2.7)	21 (6.3)	7 (6.5)	14 (4.4)	0.52
Normal	74 (39.4)	123 (37.2)	60 (55.6)	132 (41.1)	
Overweight	82 (43.6)	135 (40.8)	30 (27.8)	130 (40.5)	
Obese	27 (14.4)	52 (15.7)	11 (10.2)	45 (14.0)	
**Age (years)**	78 (73–82)	59 (54–63)	34 (27–40)	75 (71–80)	** < 0.001**
≤ 50	0 (0.0)	46 (13.9)	108 (100.0)	0 (0.0)	** < 0.001**
51–65	11 (5.9)	268 (81.0)	0 (0.0)	0 (0.0)	
66–80	115 (61.2)	17 (5.1)	0 (0.0)	241 (75.1)	
> 80	62 (33.0)	0 (0.0)	0 (0.0)	80 (24.9)	
**Department**					
Neuro	92 (48.9)	145 (43.8)	61 (56.5)	136 (42.4)	**0.001**
Surgical	72 (38.3)	152 (45.9)	40 (37.0)	164 (51.1)	
Medical	21 (11.2)	22 (6.6)	6 (5.6)	8 (2.5)	
Other	3 (1.6)	12 (3.6)	1 (0.9)	13 (4.0)	
**Hospital admission**					
From home	110 (58.5)	216 (65.3)	76 (70.4)	211 (65.7)	**0.001**
From hospital	65 (34.6)	113 (34.1)	31 (28.7)	104 (32.4)	
From nursing home	12 (6.4)	2 (0.6)	0	4 (1.2)	
Unknown	1 (0.5)	0	1 (0.9)	2 (0.6)	
**Scorings**					
Frailty	101 (53.7)	44 (13.3)	13 (12.0)	88 (27.4)	** < 0.001**
Glasgow Coma Scale	6 [3–10]	14 [7–15]	14 [7–15]	15 [14, 15]	** < 0.001**
APACHE 2	24 [20–28]	14 [9–17]	9 [4–14]	13 [10–15]	** < 0.001**
SOFA	10 [7–12]	6 [4–8]	5 [3–8]	6 [4–7]	** < 0.001**
Charlson Comorbidity Index	2 [1–4]	1 [0–2]	0 [0–1]	2 [1–3]	** < 0.001**
**ICU admission reasons**					
Sepsis	48 (25.5)	39 (11.8)	11 (10.2)	39 (12.1)	** < 0.001**
Polytrauma	2 (1.1)	8 (2.4)	18 (16.7)	7 (2.2)	** < 0.001**
Traumatic brain injury	22 (11.7)	28 (8.5)	25 (23.1)	34 (10.6)	** < 0.001**
Non-traumatic brain injury	46 (24.5)	90 (27.2)	28 (25.9)	52 (16.2)	**0.006**
Postoperative care	19 (10.1)	68 (20.5)	19 (17.6)	84 (26.2)	** < 0.001**
Cardiac failure	18 (9.6)	16 (4.8)	2 (1.9)	18 (5.6)	**0.034**
Respiratory failure	62 (33.0)	90 (27.2)	20 (18.5)	117 (36.4)	**0.002**
Other admission reasons	18 (9.6)	63 (19.0)	9 (8.3)	63 (19.6)	**0.001**
MTB at hospital admission	30 [15–30]	30 [30–30]	30 [30–30]	30 [30–30]	** < 0.001**
0	3 (1.6)	4 (1.2)	2 (1.9)	0 (0.0)	
5	13 (6.9)	2 (0.6)	2 (1.9)	2 (0.6)	
10	11 (5.9)	2 (0.6)	5 (4.6)	3 (0.9)	
15	21 (11.2)	6 (1.8)	1 (0.9)	9 (2.8)	** < 0.001**
20	24 (12.8)	8 (2.4)	1 (0.9)	12 (3.7)	
25	19 (10.1)	11 (3.3)	1 (0.9)	29 (9.0)	
30	97 (51.6)	298 (90.0)	96 (88.9)	266 (82.9)	

Data is presented as *n* (%) or median (interquartile range). Frailty is assumed at Clinical Frailty Scale ≥ 5. A *p*-value of < 0.05 was considered significant

*APACHE* Acute physiology and chronic health evaluation score, *SOFA* Sepsis-related organ failure assessment score, *MTB* Mobility-Transfer-Barthel [32]

### Mobilization parameters of the different cluster

Each parameter used to characterize mobilization differed significantly between the clusters (Table 2). Early mobilization (within the first 72 h after ICU admission) was applied to all clusters (Table 2). The dosage of mobilization differed significantly between the clusters. Patients of the cluster “Old non-frail” accomplished the longest mean daily duration (28 min [9–67 min]), those of the cluster “Young Trauma” achieved the highest maximum SOMS level (3 [2–4]), those of cluster “Severely ill & Frail” the lowest maximum SOMS level (2 [1–3]) and had the shortest mean duration of their mobilization session per day (6 min [2–27 min]. Univariate and post hoc analyses are presented in Additional file 1: Table S3.

**Table 2:** Mobilization parameters in the four clusters

	Severely ill & Frail	Middle-aged	Young Trauma	Old non-frail	*P* value
	(*N* = 188)	(*N* = 331)	(*N* = 108)	(*N* = 321)	
Frequency of mobilization session per day	1.1 (1.0–1.2)	1.2 (1.0–1.4)	1.2 (1.0–1.5)	1.2 (1.0–1.4)	** < 0.001**
Mean duration per day (min)	6 (2–27)	13 (5–39)	20 (7–51)	28 (9–67)	** < 0.001**
First day of mobilization	4 (3–7)	3 (2–7)	3 (2–7)	2 (2–4)	** < 0.001**
Early mobilization	79 (42.0)	187 (56.5)	62 (57.4)	233 (72.6)	** < 0.001**
Maximum SOMS level	2 (1–3)	3 (2, 3)	3 (2–4)	3 (2–3)	** < 0.001**
*0*	7 (3.7)	9 (2.7)	0	4 (1.2)	** < 0.001**
*1*	67 (35.6)	67 (20.2)	10 (9.3)	35 (10.9)	
*2*	61 (32.4)	82 (24.8)	26 (24.1)	91 (28.3)	
*3*	34 (18.1)	89 (26.9)	35 (32.4)	126 (39.3)	
*4*	19 (10.1)	84 (25.4)	37 (34.3)	65 (20.2)	

Data is presented as *n* (%) or median (interquartile range). Early mobilization is given, if the first session was performed earlier than 72h after ICU admission. A *p*-value of < 0.05 was considered significant. *SOMS* Surgical Intensive Care Unit Optimal Mobilization Score (ranging from 0 to 4) [18–20]

### Primary outcome

**Fig. 3:**
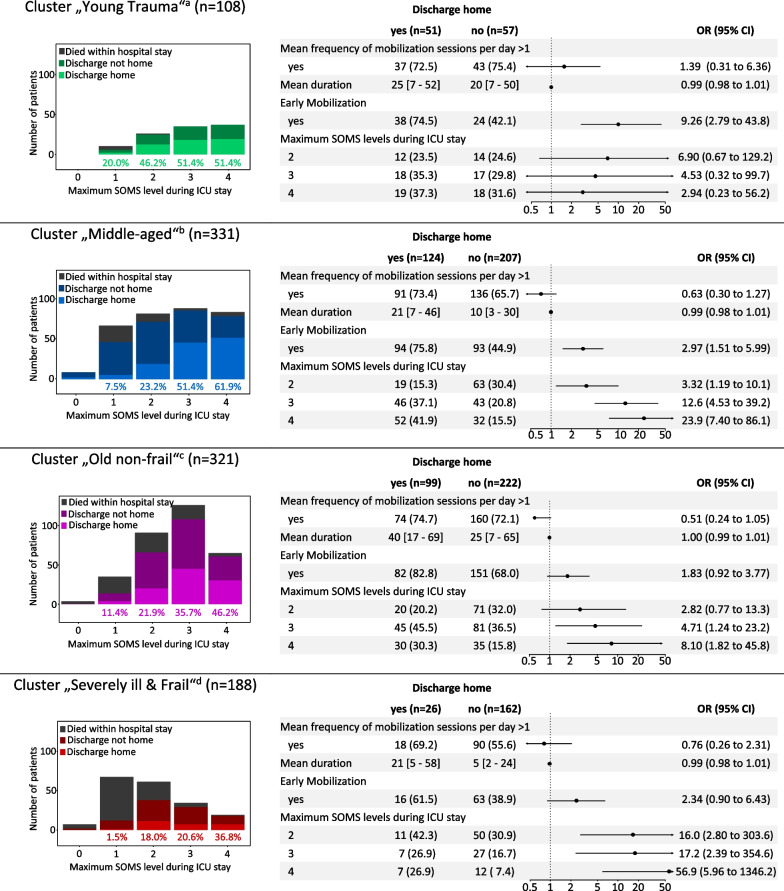
Synthetic figure summarizing the main findings. The bar charts show the number of patients in each maximum achieved SOMS level according to their discharge disposition. The percentages below the columns show the frequency of patients discharged home of each SOMS level. Numbers are presented as n (%) or median [IQR]. Early mobilization is defined as mobilization within the first 72 h after ICU admission. The reference for early mobilization is “No Early Mobilization,” the reference for maximum SOMS level achieved is “0/1″. ^a^Model was corrected for “Hospital admission,” “Body Mass Index (categories),” “Clinical Frailty Scale,” “Other ICU admission reasons,” “Postoperative care” and “SOFA.” ^b^Model was corrected for “Hospital admission,” “APACHE,” “Body Mass Index (categories),” “Charlson Comorbidity Index,” “Clinical Frailty Scale,” “Other ICU admission reasons” and “SOFA.” ^c^Model was corrected for “Hospital admission,” “Age (categories),” “APACHE,” “Mobility-Transfer-Barthel” and “Department.” ^d^Model was corrected for “Clinical Frailty Scale” and “Postoperative care.” *ICU* Intensive care unit, *IQR* Interquartile range, SOMS-Score Surgical Intensive Care Unit Optimal Mobilization Score [18–20]

The influence of mobilization on the discharge disposition home differed significantly between the clusters. Early mobilization (< 72 h) was the most significant factor in the cluster “Young Trauma” (OR_adj_ 10.0 [2.8 to 44.0], *p* < 0.001) and in the cluster of “Middle-aged” patients (cluster 2, OR_adj_ 3.0 (95%CI [1.5 to 6.0]), *p* < 0.001), whereas there was no significant influence in the other clusters (Fig. 3). In the clusters “Middle-aged” and “Severely ill & Frail,” the likelihood of being discharged home increased with each SOMS level achieved, up to a 24-fold increased likelihood with SOMS level 4 (OR_adj_ 23.9 [7.4 to 86.1], *p* < 0.001 and OR_adj_ 56.9 [6.9 to 1346.2], *p* = 0.002, respectively). In the cluster “Old non-frail,” the probability to be discharged home increased significantly, if SOMS > 3 was achieved (OR_adj_ 4.7 [1.2 to 23.2], *p* = 0.035 and OR_adj_ 8.1 [1.8 to 45.8], *p* = 0.010, respectively) (Fig. 3). The logistic regression models for possible confounders are given in Additional file 1: Table S4, the sensitivity analysis with survivors only in Additional file 1: Table S5 and the 11-point IMS results that confirm the results of the primary analysis in Additional file 1: Table S6.

### Secondary outcomes

The probability to be discharged home as well as hospital mortality differed significantly between the clusters in the univariate analysis (Fig. 3 and Additional file 1: Table S7). ICU and hospital mortality were the highest in the cluster “Severely ill & Frail” (34% and 42%, *p* < 0.001, respectively), whereas in the cluster “Young Trauma” mortality was only 1.1% (for details Additional file 1: Table S7).

## Discussion

In this study, critically ill patients were homogeneously clustered with their clinical and procedural characteristics to evaluate the personalized benefits of the components of mobilization therapy for discharge home using a machine learning technology. The resulting four clusters (“Severely ill & Frail,” “Middle-aged,” “Young Trauma” and “Old non-frail”) differed in the components of mobilization and in the frequency of discharge home. In the clusters “Young Trauma” and “Middle-aged,” early mobilization was strongly associated with discharge home. In the clusters “Middle-aged,” “Old non-frail” and “Severely ill & Frail,” the achieved level of mobilization during ICU stay indicated the best chance of discharge home.

While studies have shown that early mobilization improves short-term patient outcomes [3–5, 36], the individual optimal dose of mobilization is unknown. Using the FITT principle, the mobilization dose can be specified by frequency, intensity, time and modality [10, 37]. Whereas reporting of these components was mostly incomplete across studies, in our prospectively designed observational database, they were explicitly queried.

Since there is still no uniform definition when to start early mobilization [38], we used definition of the German guideline, i.e., start within 72 h of ICU admission, which was also confirmed beneficial in a network-metanalysis [25, 39]. While early mobilization was performed in all four clusters, a significant effect on outcome was only evident in the clusters of “Young Trauma” and “Middle-aged” patients.

There is no evidence on the optimal frequency of mobilization. In their prospective, randomized clinical trial, Winkelmann et al. found no benefit of two mobilization sessions compared to one [40]. In accordance, Paton et al. also found that health six months after ICU therapy was not better when patients were treated with more mobilization sessions per day [15]. Also, Scheffenbichler et al. concluded in their investigation of surgical critically ill patients that level and duration were modifying outcome but not frequency [9]. In our study, however, patients of the cluster “Old non-frail” benefited from more frequent mobilization sessions. As is known from stroke patients, elderly patients may benefit from more frequent but shorter sessions thus avoiding overuse given their lower physiological reserves [41].

Most important for the patients seemed to be the achieved level of mobilization in the ICU. Except for the “Young Trauma” cluster, all other clusters benefited significantly from higher levels of mobilization. Especially in the cluster “Severely ill & Frail,” patients achieving SOMS levels ≥ 2 (sitting) had lower mortality and a higher chance of being discharged home. These findings are aligned with all other studies investigating level. Paton et al. [15] and also Scheffenbichler et al. [9] found that the ability to stand (SOMS ≥ 3) in the ICU was an important milestone for improving 6-month outcomes. Dos Santos Moraes et al. showed that patients, who achieved high IMS scores, had significantly increased likelihood of being discharged home and a reduced probability of in-hospital death. Unfortunately, the authors did not include data of prehospital functional status or comorbidities in their analysis [14].

This shortcoming was addressed by Mayer et al., who demonstrated that ECMO patients achieving higher mobility levels had a better chance to survive [42]. However, the authors raised the question of whether early mobilization actually improves outcome and whether the more favorable disease course does not allow mobilization with more rapid progression to higher levels [42]. Therefore, we adjusted mobilization parameters for disease severity, age and other influencing factors. Importantly, mobilization results were not altered by these adjustments, underscoring the relevance of this intervention.

Most interestingly duration did not modify the effect on any of our clusters which is also important for resource allocation. In contrast, Scheffenbichler et al. demonstrated that duration had a positive impact [9]. The authors pointed out that their findings contrast with those of stroke patients who were treated in stroke units but did not require intensive care. Here, the length of mobilization in particular had a negative effect on outcome if the sessions were not split up in several short sessions [41]. Although our unsupervised learning approach did not identify a cluster of exclusively neurocritical patients, they were included in this study. Nevertheless, duration of mobilization should be investigated in future studies.

In summary, for each of the four clusters, different effect modifications of mobilization components on discharge disposition could be demonstrated. This emphasizes the relevance of subdividing the heterogeneous cohort of critically ill patients, e.g., by functionality and pre-existing frailty or independence.

We recognize some key limitations. Generalizability is limited as a single-center study. However, the aim of this study was feasibility of an individual approach to mobilization. External validation will be a future aim. Of particular importance here is the extent, to which a causality between mobilization and outcome can be confirmed or whether the outcome is driven by patient characteristics. However, by adjusting the models for disease severity, age and previous health conditions, the clusters are homogeneous with respect to these factors. Although an even finer differentiation might provide more individualization, it would have resulted in very small clusters that are difficult to define clinically. Third, a differentiation of the modality of mobilization as described in the FITT principle was not part of our analysis. However, mobilization was recorded independent of the executing staff (e.g., physiotherapy, nurses, doctors) with details of timing, level and duration. In addition, barriers to mobilization or discontinuation criteria were not recorded in this study.

## Conclusion

Using machine learning, an identification of defined patient clusters was possible. These four clusters (“Severely ill & Frail,” “Middle-aged,” “Young Trauma” and “Old non-frail”) had different clinical characteristics. Furthermore, different mobilization components were important for the respective cluster's outcome. After external and prospective evaluation, this clustering may allow to individualize mobilization of critically ill patients and improve outcome. Competing risk analyses including time-dependent variables may further help to understand how the trajectory of intensive care and the course of mobilization interact with respect to outcome.

## Supplementary Information

**Additional file 1. Figure S1:** Elbow method for determination of clusters. **Figure S2: Euclidian distance plot.** Heatmap showing Euclidean distances between samples clustered using complete linkage. Factors used for Cluster Analysis are visualized as column annotations. **Table S1: Patient Characteristics.** Numbers are presented as *n* (%) or median [IQR]. “Frail” is defined as Clinical Frailty Scale 5–9. A *p*-value of < 0.05 was considered significant. *ICU* Intensive Care Unit, *IQR* Interquartile Range, *APACHE II* Acute Physiology and Chronic Health Evaluation Score, *SOFA* Sepsis-related Organ Failure Assessment Score, *MTB* Mobility-Transfer-Barthel. **Table S2: Post-hoc Analyses of patient characteristics in the four clusters.** Numbers are presented as *n* (%) or median [IQR]. “Frail” is defined as Clinical Frailty Scale 5–9. A *p*-value of < 0.05 was considered significant. *APACHE* Acute Physiology and Chronic Health Evaluation Score, *SOFA* Sepsis-related Organ Failure Assessment Score, *MTB* Mobility-Transfer-Barthel. **Table S3: Post-hoc Analyses of mobilization characteristics in the four clusters.** Early mobilization is defined as mobilization within 72h of ICU admission. A *p*-value of < 0.05 was considered significant. Numbers are presented as *n* (%) or median [IQR]. *IQR* Interquartile Range, *SOMS* Surgical Intensive Care Unit Optimal Mobilization Score. **Table S4: Stepwise Regression Models.** “Frailty” is defined as Clinical Frailty Scale 5–9. A *p*-value of < 0.05 was considered significant. Early mobilization is defined as mobilization within 72h of ICU admission. *OR* Odds Ratio, *CI* Confidence interval, *ICU* Intensive Care Unit, *IQR* Interquartile Range, *APACHE II* Acute Physiology and Chronic Health Evaluation Score, *SOFA* Sepsisrelated Organ Failure Assessment Score, *SOMS* Surgical Intensive Care Unit Optimal Mobilization Score. **Table S5: Logistic regression models of survivors of hospital stay.** Early mobilization is defined as mobilization within 72h of ICU admission. A *p*-value of < 0.05 was considered significant. *OR* Odds Ratio, *CI* Confidence interval, *SOMS* Surgical Intensive Care Unit Optimal Mobilization Score. **a** Model was corrected for postoperative care, frailty, department. **b** Model was corrected for Sepsis-related Organ Failure Assessment Score, body mass index (categories), postoperative, frailty, other admission reasons, admission, and non-traumatic brain injury. **c** Model was corrected for department and Glasgow Coma Scale. **d** Model was corrected for APACHE II, department, admission, Mobility-Transfer-Barthel Score at hospital admission and age (categories). **Table S6: Sensitivity Analysis with the ICU Mobility Scale.** Early mobilization is defined as mobilization within 72h of ICU admission. A *p*-value of < 0.05 was considered significant. *OR* Odds Ratio, *CI* Confidence interval. *IQR* Interquartile Range, *IMS* ICU Mobility Scale. **Table S7: Secondary Endpoints.** Secondary endpoints in dependence of the four clusters. Here, the results of the primary analysis as well as the post-hoc analyses are listed. Numbers are presented as *n* (%) or median [IQR]. A *p*-value of < 0.05 was considered significant. *IQR* Interquartile Range, *ICU* Intensive Care Unit.

## Data Availability

The datasets used and/or analyzed during the current study are available from the corresponding author on reasonable request.
