# Supplementary material for: Clustering of critically ill patients using an individualized learning approach enables dose optimization of mobilization in the ICU
Source: Crit Care. 2023 Jan 3;27:1. doi: 10.1186/s13054-022-04291-8 (PMC9808956; doi:10.1186/s13054-022-04291-8)
Supplement: Supplementary file 1 — Additional file 1. Figure S1: Elbow method for determination of clusters. Figure S2: Euclidian distance plot. Heatmap showing Euclidean distances between samples clustered using complete linkage. Factors used for Cluster Analysis are visualized as column annotations. Table S1: Patient Characteristics. Numbers are presented as n (%) or median [IQR]. “Frail” is defined as Clinical Frailty Scale 5–9. A p-value of < 0.05 was considered significant. ICU Intensive Care Unit, IQR Interquartile Range, APACHE II Acute Physiology and Chronic Health Evaluation Score, SOFA Sepsis-related Organ Failure Assessment Score, MTB Mobility-Transfer-Barthel. Table S2: Post-hoc Analyses of patient characteristics in the four clusters. Numbers are presented as n (%) or median [IQR]. “Frail” is defined as Clinical Frailty Scale 5–9. A p-value of < 0.05 was considered significant. APACHE Acute Physiology and Chronic Health Evaluation Score, SOFA Sepsis-related Organ Failure Assessment Score, MTB Mobility-Transfer-Barthel. Table S3: Post-hoc Analyses of mobilization characteristics in the four clusters. Early mobilization is defined as mobilization within 72h of ICU admission. A p-value of < 0.05 was considered significant. Numbers are presented as n (%) or median [IQR]. IQR Interquartile Range, SOMS Surgical Intensive Care Unit Optimal Mobilization Score. Table S4: Stepwise Regression Models. “Frailty” is defined as Clinical Frailty Scale 5–9. A p-value of < 0.05 was considered significant. Early mobilization is defined as mobilization within 72h of ICU admission. OR Odds Ratio, CI Confidence interval, ICU Intensive Care Unit, IQR Interquartile Range, APACHE II Acute Physiology and Chronic Health Evaluation Score, SOFA Sepsisrelated Organ Failure Assessment Score, SOMS Surgical Intensive Care Unit Optimal Mobilization Score. Table S5: Logistic regression models of survivors of hospital stay. Early mobilization is defined as mobilization within 72h of ICU admission. A p-value of < 0.05 was c [file 13054_2022_4291_MOESM1_ESM.pdf]

Additional File 1

“Clustering of critically ill patients using an individualized learning approach enables dose optimisation of mobilization in the ICU”

ADDITIONAL FILE – FIGURES 2

ADDITIONAL FILE FIGURE S1: ELBOW METHOD FOR DETERMINATION OF CLUSTERS. 2
ADDITIONAL FILE FIGURE S2: EUCLIDIAN DISTANCE PLOT. 3

ADDITIONAL FILE – TABLES 4

ADDITIONAL FILE TABLE S1: PATIENT CHARACTERISTICS. 4
ADDITIONAL FILE TABLE S2: POST-HOC ANALYSES OF PATIENT CHARACTERISTICS IN THE FOUR CLUSTERS. 5
ADDITIONAL FILE TABLE S3: POST-HOC ANALYSES OF MOBILIZATION CHARACTERISTICS IN THE FOUR CLUSTERS. 6
ADDITIONAL FILE TABLE S4: STEPWISE REGRESSION MODELS. 7
ADDITIONAL FILE TABLE S5: LOGISTIC REGRESSION MODELS OF SURVIVORS OF HOSPITAL STAY. 9
ADDITIONAL FILE TABLE S6: SENSITIVITY ANALYSIS WITH THE ICU MOBILITY SCALE. 10
ADDITIONAL FILE TABLE S7: SECONDARY ENDPOINTS. 11

Additional File – Figures

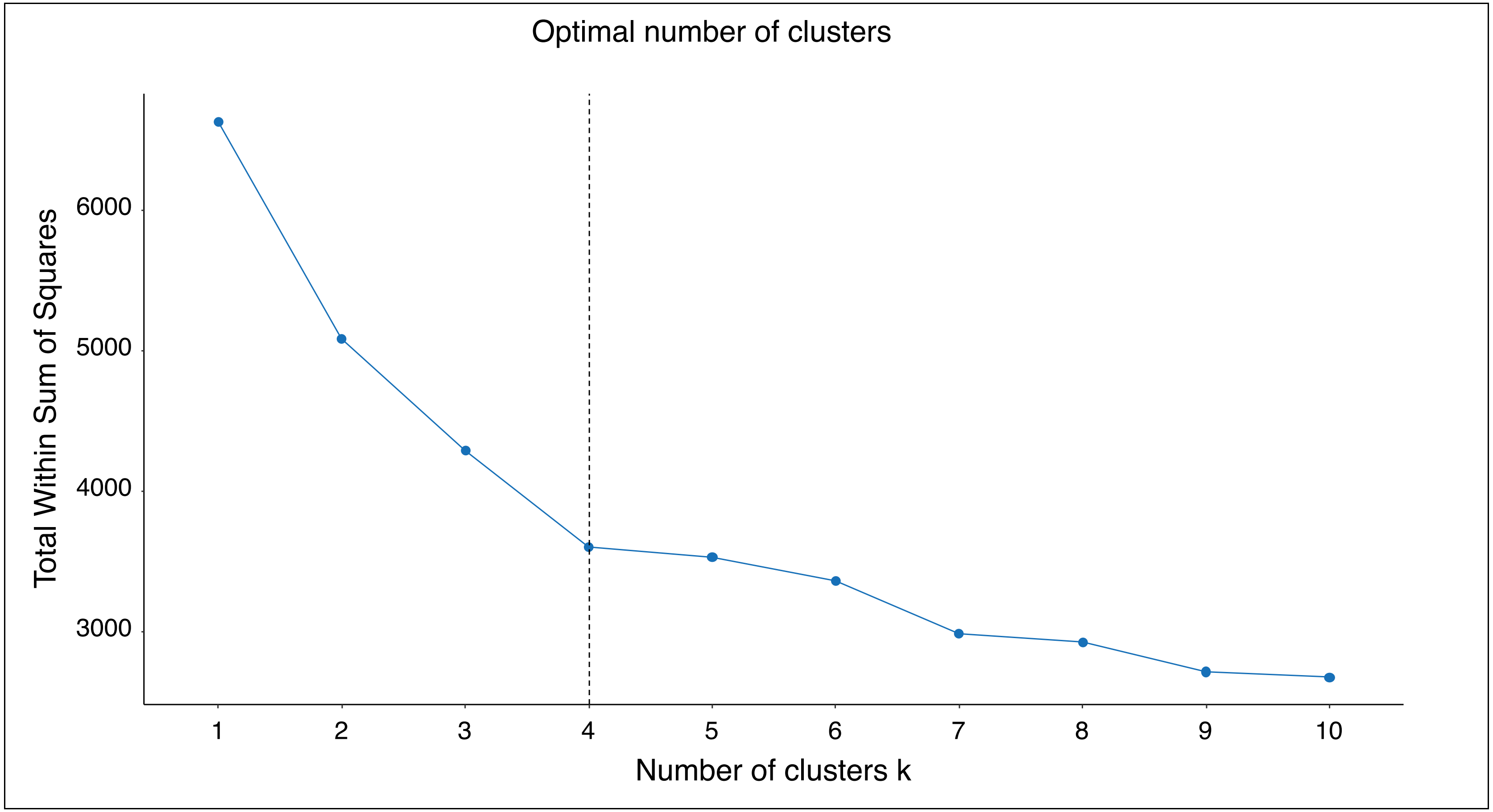

Additional File Figure S1: Elbow method for determination of clusters.

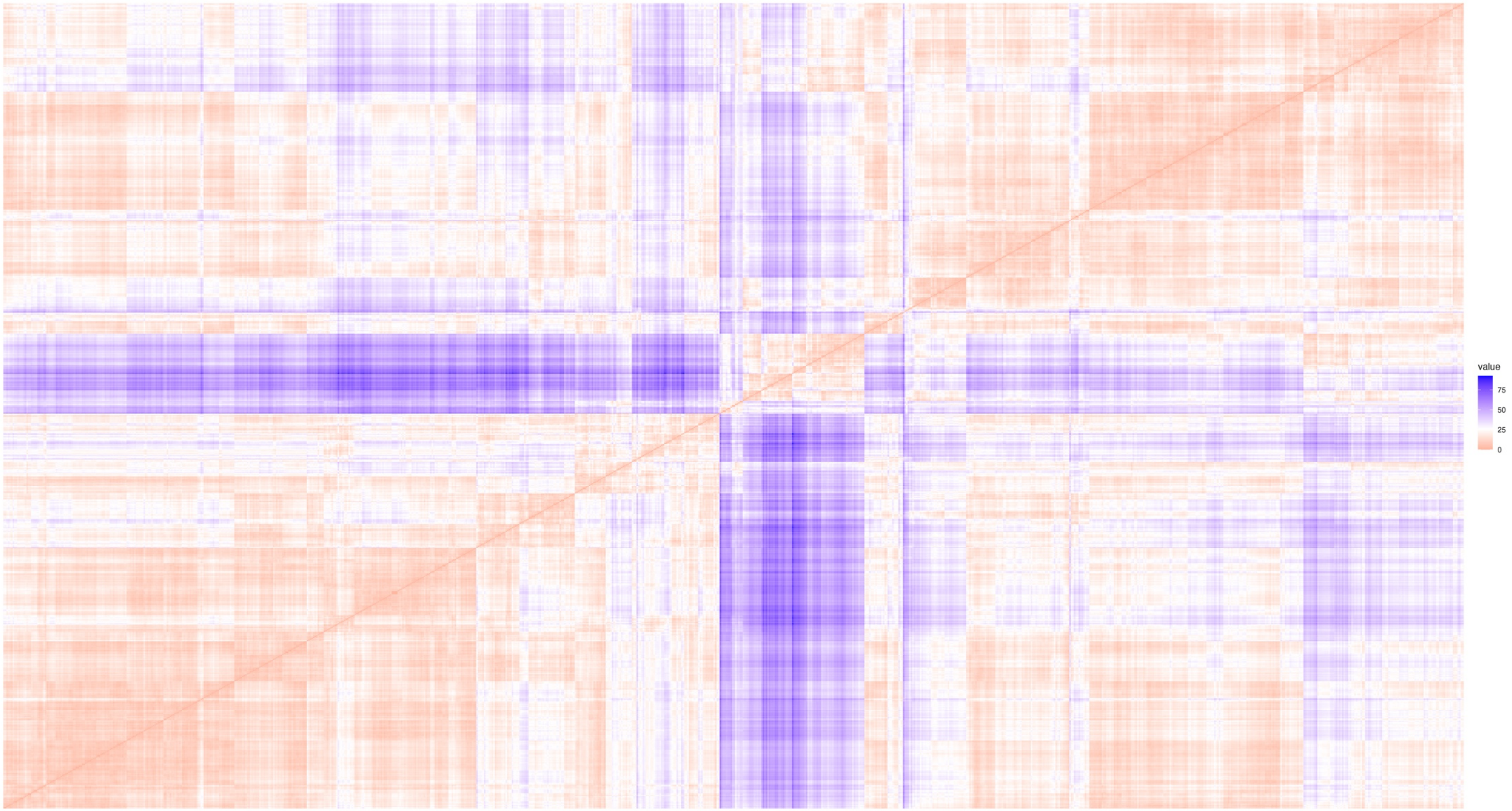

Additional File Figure S2: Euclidian distance plot.
Heatmap showing Euclidean distances between samples clustered using complete linkage. Factors used for Cluster Analysis are visualized as column annotations.

Additional File – Tables

Additional File Table S1: Patient Characteristics.

	Discharge home	
	**no,** N = 648	**yes,** N = 300
Female sex	378 (58.3)	176 (58.7)
Body Mass Index (kg/m2)	25.4 [23.1 – 27.7]	25.7 [23.1 – 28.5]
**Body Mass Index (kg/m2)**		
underweight	23 (3.5)	24 (8.0)
normal	281 (43.4)	108 (36.0)
overweight	264 (40.7)	113 (37.7)
obese	80 (12.3)	55 (18.3)
Age (years)	70 [59 – 78]	62 [53 – 72]
**Age (years)**		
≤50	88 (13.6)	66 (22.0)
51-65	173 (26.7)	106 (35.3)
66-80	264 (40.7)	109 (36.3)
>80	123 (19.0)	19 (6.3)
**Department**		
Neuro	346 (53.4)	88 (29.3)
Surgical	249 (38.4)	179 (59.7)
Medical	40 (6.2)	17 (5.7)
Other admission reasons	13 (2.0)	16 (5.3)
**Admission**		
from home	383 (59.1)	230 (76.7)
from hospital	245 (37.8)	68 (22.7)
from nursing home	17 (2.6)	1 (0.3)
unknown	3 (0.5)	1 (0.3)
Ventilated during the ICU stay	425 (65.6)	125 (41.7)
**Scoring**		
Frailty	207 (31.9)	39 (13.0)
Glasgow Coma Scale	13 [6 – 15]	15 [13 – 15]
APACHE 2	16 [11 – 20]	12 [8 – 15]
SOFA	7 [5 – 9]	5 [3 – 7]
Charlson Comorbidity Index	2 [0 – 3]	1 [0 – 2]
**Admission reason**		
Sepsis	107 (16.5)	30 (10.0)
Polytrauma	24 (3.7)	11 (3.7)
Traumatic brain Injury	86 (13.3)	23 (7.7)
Postoperative Care	88 (13.6)	102 (34.0)
Cardiac Failure	41 (6.3)	13 (4.3)
Respiratory Failure	208 (32.1)	81 (27.0)
Other admission reasons	92 (14.2)	61 (20.3)
**Pre-morbid functional status**		
MTB at hospital admission	30 [30 – 30]	30 [30 – 30]
MTB at hospital admission		
0	7 (1.1)	2 (0.7)
5	17 (2.6)	2 (0.7)
10	19 (2.9)	2 (0.7)
15	30 (4.6)	7 (2.3)
20	34 (5.2)	11 (3.7)
25	40 (6.2)	20 (6.7)
30	501 (77.3)	256 (85.3)

Numbers are presented as n (%) or median [IQR]. “Frail” is defined as Clinical Frailty Scale 5-9. *ICU* Intensive Care Unit, *IQR* Interquartile Range, *APACHE II* Acute Physiology and Chronic Health Evaluation Score, *SOFA* Sepsis-related Organ Failure Assessment Score, *MTB* Mobility-Transfer-Barthel

Additional File Table S2: Post-hoc Analyses of patient characteristics in the four clusters.

	Cluster				Post-hoc Analyses					
	**Severely ill & Frail, (N = 188)**	**Middle-aged, (N = 331)**	**Young Trauma, (N = 108)**	**Old non-frail, (N = 321)**	Severely ill & Frail vs. Middle-aged	Severely ill & Frail vs. Young Trauma	Severely ill & Frail vs. Old non-frail	Middle-aged vs. Young Trauma	Middle-aged vs. Old non-frail	Young Trauma vs. Old non-frail
Female sex	107 (56.9)	202 (61.0)	60 (55.6)	185 (57.6)						
Body mass index (kg/m2)	25.4 [23.5 – 27.7]	25.7 [23.1 – 28.1]	24.2 [21.1 – 26.7]	25.4 [23.3 – 28.1]	>0.9	0.009	0.010	>0.9	>0.9	0.019
**Body mass index (kg/m2)**										
underweight	5 (2.7)	21 (6.3)	7 (6.5)	14 (4.4)						
normal	74 (39.4)	123 (37.2)	60 (55.6)	132 (41.1)						
overweight	67 (35.6)	127 (38.4)	29 (26.9)	114 (35.5)						
obese	27 (14.4)	52 (15.7)	11 (10.2)	45 (14.0)						
Age (years)	78 [73 – 82]	59 [54 – 63]	34 [27 – 40]	75 [71 – 80]	<0.001	<0.001	0.2	<0.001	<0.001	<0.001
**Age (years)**					0.003	0.003	0.003	0.003	0.003	0.003
≤50	0 (0.0)	46 (13.9)	108 (100.0)	0 (0.0)						
51-65	11 (5.9)	268 (81.0)	0 (0.0)	0 (0.0)						
66-80	115 (61.2)	17 (5.1)	0 (0.0)	241 (75.1)						
>80	62 (33.0)	0 (0.0)	0 (0.0)	80 (24.9)						
**Department**					0.5	>0.9	0.003	0.6	0.3	0.057
Neuro	92 (48.9)	145 (43.8)	61 (56.5)	136 (42.4)						
Surgical	72 (38.3)	152 (45.9)	40 (37.0)	164 (51.1)						
Medical	21 (11.2)	22 (6.6)	6 (5.6)	8 (2.5)						
Other admission reasons	3 (1.6)	12 (3.6)	1 (0.9)	13 (4.0)						
Neurocritical	92 (48.9)	145 (43.8)	61 (56.5)	136 (42.4)						
**Admission**					0.003	0.063	0.033	>0.9	>0.9	>0.9
from home	110 (58.5)	216 (65.3)	76 (70.4)	211 (65.7)						
from hospital	65 (34.6)	113 (34.1)	31 (28.7)	104 (32.4)						
from nursing home	12 (6.4)	2 (0.6)	0 (0.0)	4 (1.2)						
unknown	1 (0.5)	0 (0.0)	1 (0.9)	2 (0.6)						
**Scorings**										
Frailty	101 (53.7)	44 (13.3)	13 (12.0)	88 (27.4)	<0.001	<0.001	<0.001	>0.9	<0.001	0.006
Glasgow Coma Scale	6 [3 – 10]	14 [7 – 15]	14 [7 – 15]	15 [14 – 15]	<0.001	<0.001	<0.001	>0.9	<0.001	<0.001
APACHE 2	24 [20 – 28]	14 [9 – 17]	9 [4 – 14]	13 [10 – 15]	<0.001	<0.001	<0.001	<0.001	>0.9	<0.001
SOFA	10 [7 – 12]	6 [4 – 8]	5 [3 – 8]	6 [4 – 7]	<0.001	<0.001	<0.001	0.082	0.070	>0.9
Charlson Comorbidity Index	2 [1 – 4]	1 [0 – 2]	0 [0 – 1]	2 [1 – 3]	<0.001	<0.001	0.8	<0.001	<0.001	<0.001
**ICU Admission Reasons**										
Sepsis	48 (25.5)	39 (11.8)	11 (10.2)	39 (12.1)	<0.001	0.008	<0.001	>0.9	>0.9	>0.9
Polytrauma	2 (1.1)	8 (2.4)	18 (16.7)	7 (2.2)	>0.9	<0.001	>0.9	<0.001	>0.9	<0.001
Traumatic brain injury	22 (11.7)	28 (8.5)	25 (23.1)	34 (10.6)	>0.9	0.077	>0.9	<0.001	>0.9	0.011
Non-traumatic brain injury	46 (24.5)	90 (27.2)	28 (25.9)	52 (16.2)	>0.9	>0.9	0.2	>0.9	0.005	0.2
Postoperative Care	19 (10.1)	68 (20.5)	19 (17.6)	84 (26.2)	0.013	0.4	<0.001	>0.9	0.6	0.5
Cardiac Failure	18 (9.6)	16 (4.8)	2 (1.9)	18 (5.6)	0.3	0.084	0.6	>0.9	>0.9	>0.9
Respiratory Failure	62 (33.0)	90 (27.2)	20 (18.5)	117 (36.4)	>0.9	0.043	>0.9	0.4	0.070	0.003
Other admission reasons	18 (9.6)	63 (19.0)	9 (8.3)	63 (19.6)	0.023	>0.9	0.015	0.062	>0.9	0.042
**Pre-morbid functional Status**										
MTB at hospital admission	30 [15 – 30]	30 [30 – 30]	30 [30 – 30]	30 [30 – 30]	<0.001	<0.001	<0.001	>0.9	0.074	>0.9

Numbers are presented as n (%) or median [IQR]. “Frail” is defined as Clinical Frailty Scale 5-9.

APACHE Acute Physiology and Chronic Health Evaluation Score, *SOFA* Sepsis-related Organ Failure Assessment Score, *MTB* Mobility-Transfer-Barthel

Additional File Table S3: Post-hoc Analyses of mobilization characteristics in the four clusters.

	Cluster					Post-hoc Analyses					
	**Severely ill & Frail, (N = 188)**	**Middle-aged, (N = 331)**	**Young Trauma, (N = 108)**	**Old non-frail, (N = 321)**	**p value**	**Severely ill & Frail vs. Middle-aged**	**Severely ill & Frail vs. Young Trauma**	**Severely ill & Frail vs. Old non-frail**	**Middle-aged vs. Young Trauma**	**Middle-aged vs. Old non-frail**	**Young Trauma vs. Old non-frail**
Frequency of mobilization session per day	1.1 [1.0 - 1.2]	1.2 [1.0 - 1.4]	1.2 [1.0 - 1.5]	1.2 [1.0 - 1.4]	<0.001	<0.001	<0.001	>0.9	<0.001	>0.9	>0.9
Mean duration per day (minutes)	6 [2 - 27]	13 [5 - 39]	20 [7 - 51]	28 [9 - 67]	<0.001	<0.001	<0.001	0.4	<0.001	<0.001	0.5
Average SOMS level	1.5 [1.0 - 2.0]	1.9 [1.2 - 2.5]	2.0 [1.7 - 2.8]	2.0 [1.5 - 2.5]	<0.001	<0.001	<0.001	0.004	<0.001	0.2	0.3
First day of mobilization	4.0 [3.0 - 7.0]	3.0 [2.0 - 7.0]	3.0 [2.0 - 7.0]	2.0 [2.0 - 4.0]	<0.001	0.10	0.7	>0.9	<0.001	<0.001	0.005
Early mobilization	79 (42.0)	187 (56.5)	62 (57.4)	233 (72.6)	<0.001	0.012	0.058	>0.9	<0.001	<0.001	0.031
Maximum SOMS level reached	2 [1 – 3]	3 [2 – 3]	3 [2 – 4]	3 [2 – 3]	<0.001	<0.001	<0.001	0.012	<0.001	>0.9	0.10
Maximum SOMS level reached					<0.001	0.003	0.003	0.12	0.003	0.033	0.3
0	7 (3.7)	9 (2.7)	0 (0.0)	4 (1.2)							
1	67 (35.6)	67 (20.2)	10 (9.3)	35 (10.9)							
2	61 (32.4)	82 (24.8)	26 (24.1)	91 (28.3)							
3	34 (18.1)	89 (26.9)	35 (32.4)	126 (39.3)							
4	19 (10.1)	84 (25.4)	37 (34.3)	65 (20.2)							

Early mobilization is defined as mobilization within 72h of ICU admission. Numbers are presented as n (%) or median [IQR].
IQR Interquartile Range *SOMS* Surgical Intensive Care Unit Optimal Mobilization Score

Additional File Table S4: Stepwise Regression Models.

**Cluster "Severely ill & Frail"**		
	**OR (95% CI]**	**p value**
Frequency of mobilization session per day	1.32 [0.43 - 3.91]	0.62
Mean duration per day (minutes)	0.99 [0.98 - 1.01]	0.49
Early mobilization	2.34 [0.90 - 6.43]	0.09
Maximum SOMS level reached		0.001
2	16.0 [2.80 - 304]	0.010
3	17.2 [2.39 - 355]	0.014
4	56.9 [5.96 – 1.346]	0.002
Postoperative Care	5.73 [1.64 - 20.8]	0.006
Frailty	0.36 [0.12 - 0.97]	0.044
**Cluster "Middle-aged"**		
	**OR [95% CI]**	**p value**
Frequency of mobilization session per day	1.59 [0.78 - 3.26]	0.20
Mean duration per day (minutes)	0.99 [0.98 - 1.00]	0.06
Early mobilization	2.97 [1.51 - 5.99]	0.002
Maximum SOMS level reached		<0.001
2	3.32 [1.19 - 10.1]	0.027
3	12.6 [4.53 - 39.2]	<0.001
4	23.9 [7.40 - 86.1]]	<0.001
APACHE II	1.04 [0.97 - 1.12]	0.30
Frailty	0.34 [0.12 - 0.92]	0.038
Postoperative Care	6.72 [2.85 - 16.8]	<0.001
SOFA	0.81 [0.70 - 0.93]	0.002
**Body Mass Index**		0.002
Underweight	—	
Normal	0.39 [0.11 - 1.30]	0.13
Overweight	0.90 [0.28 - 2.84]	0.86
Obese	2.23 [0.61 - 8.27]	0.22
Charlson Comorbidity Score	0.85 [0.72 - 0.98]	0.025
Admission Category "Other"	2.77 [1.23 - 6.34]	0.015
Non- traumatic brain injury	2.33 [0.99 - 5.69]	0.06
**Admission From**		0.09
From home	—	
From hospital	0.49 [0.25 - 0.95]	0.036
From nursing home	0.00 [NA - 1.2e57]	0.99
**Cluster "Young Trauma"**		
	**OR [95% CI]**	**p value**
Frequency of mobilization session per day	0.72 [0.16 - 3.24]	0.67
Mean duration per day (minutes)	0.99 [0.98 - 1.01]	0.53
Early mobilization	9.98 [2.79 - 43.8]	<0.001
Maximum SOMS level reached		0.39
2	6.90 [0.67 – 129]	0.14
3	4.53 [0.32 – 99.7]	0.30
4	2.94 [0.23 – 56.2]	0.43
Non-traumatic brain injury	0.30 [0.06 – 1.43]	0.14
**Admission From**		0.002
From home	—	
From hospital	0.30 [0.07 – 1.20]	0.10
ICU Admission Category "Cardiac Failure"	730,481,516 [0.00 – NA]	>0.99
SOFA	0.78 [0.61 – 0.97]	0.022
**Body Mass Index**		0.011
Underweight	—	
Normal	0.12 [0.00 – 2.13]	0.20
Overweight	0.08 [0.00 – 1.57]	0.14
Obese	1.83 [0.03 – 74.0]	0.75
**Department**		0.036
Neuro	—	
Surgical	2.54 [0.61 – 11.6]	0.21
Medical	1.15 [0.07 – 33.2]	0.92
Other admission reasons	1,306,641,486 [0.00 – NA]	>0.99
Gender Male	3.44 [1.01 – 13.3]	0.06
Frailty	0.19 [0.02 – 1.20]	0.09

Cluster “Old non-frail”		
	**OR [95% CI]**	**p value**
Frequency of mobilization session per day	1.96 [0.96 – 4.08]	0.07
Mean duration per day (minutes)	1.00 [0.99 – 1.01]	0.71
Early mobilization	1.83 [0.92 – 3.77]	0.09
Maximum SOMS level reached		0.034
2	2.82 [0.77 - 13.3]	0.15
3	4.71 [1.24 - 23.2]	0.035
4	8.10 [1.82 - 45.8]	0.010
APACHE II	0.83 [0.76 - 0.91]	<0.001
**Department**		0.011
Neuro	—	
Surgical	1.83 [0.96 - 3.54]	0.07
Medical	12.2 [1.94 - 71.9]	0.005
Other admission reasons	4.30 [1.08 - 17.9]	0.039
**Age**		0.06
66-80	—	
>80	0.50 [0.23 - 1.04]	0.07
Frailty	0.40 [0.19 - 0.82]	0.014
Mobility-Transfer-Barthel at hospital admission	0.91 [0.84 - 0.97]	0.006
**Admission From**		0.08
From home	—	
From hospital	0.54 [0.28 - 1.04]	0.07
From nursing home	0.00 [NA - 3.1e25]	0.98
Unknown	2.04 [0.05 - 78.2]	0.68
Postoperative Care	1.64 [0.88 - 3.07]	0.12

“Frailty” is defined as Clinical Frailty Scale 5-9. Early mobilization is defined as mobilization within 72h of ICU admission. *OR* Odds Ratio, *CI* Confidence interval, *ICU* Intensive Care Unit, *IQR* Interquartile Range, *APACHE II* Acute Physiology and Chronic Health Evaluation Score, *SOFA* Sepsis-related Organ Failure Assessment Score, SOMS Surgical Intensive Care Unit Optimal Mobilization Score

Additional File Table S5: Logistic regression models of survivors of hospital stay.

**Cluster "Severely ill & Frail"**	**Only mobilization multivariate**		**Multivariate adjusted[a]**	
	OR [95% CI]	p-value	OR [95% CI]	p-value
Frequency of mobilization session per day	1.11 [0.36 - 3.25]	0.85	1.31 [0.38 - 4.44]	0.67
Mean duration per day (minutes)	0.99 [0.97 - 1.00]	0.13	0.99 [0.97 - 1.01]	0.45
Early mobilization	1.97 [0.74 - 5.58]	0.18	2.51 [0.80 - 8.37]	0.12
Maximum SOMS level reached		0.11		0.25
2	5.03 [0.79 - 98.8]	0.15	3.71 [0.52 - 76.3]	0.26
3	5.38 [0.69 - 115]	0.16	2.39 [0.23 - 57.4]	0.50
4	15.3 [1.61 - 362]	0.033	7.65 [0.66 - 199]	0.13
**Cluster "Middle-aged"**	**Only mobilization multivariate**		**Multivariate adjusted[b]**	
	OR [95% CI]	p-value	OR [95% CI]	p-value
Frequency of mobilization session per day	1.58 [0.83 - 3.06]	0.17	1.59 [0.77 - 3.35]	0.22
Mean duration per day (minutes)	0.99 [0.99 - 1.00]	0.21	0.99 [0.98 - 1.00]	0.066
Early mobilization	2.91 [1.67 - 5.16]	<0.001	2.69 [1.34 - 5.47]	0.006
Maximum SOMS level reached		<0.001		<0.001
2	2.70 [1.04 - 7.70]	0.049	2.34 [0.82 - 7.29]	0.12
3	8.22 [3.23 - 23.4]	<0.001	7.92 [2.80 - 24.9]	<0.001
4	16.7 [5.92 - 52.4]	<0.001	16.5 [4.97 - 60.9]	<0.001
**Cluster "Young Trauma"**	**Only mobilization multivariate**		**Multivariate adjusted[c]**	
	OR [95% CI]	p-value	OR [95% CI]	p-value
Frequency of mobilization session per day	1.22 [0.38 - 4.05]	0.74	0.62 [0.16 - 2.34]	0.48
Mean duration per day (minutes)	0.99 [0.98 - 1.01]	0.37	0.99 [0.98 - 1.01]	0.29
Early mobilization	6.08 [2.42 - 16.5]	<0.001	5.31 [1.88 - 16.5]	0.002
Maximum SOMS level reached		0.87		0.81
2	1.30 [0.19 – 11.8]	0.79	1.00 [0.11 – 13.7]	>0.99
3	1.05 [0.13 – 10.1]	0.96	0.47 [0.04 – 7.21]	0.57
4	1.59 [0.21 – 14.9]	0.66	0.59 [0.05 – 8.44]	0.68
**Cluster "Old non-frail"**	**Only mobilization multivariate**		**Multivariate adjusted[d]**	
	OR [95% CI]	p-value	OR [95% CI]	p-value
Frequency of mobilization session per day	1.43 [0.72 – 2.82]	0.30	1.67 [0.79 – 3.55]	0.18
Mean duration per day (minutes)	1.00 [0.99 – 1.01]	0.68	1.00 [0.99 – 1.01]	0.78
Early mobilization	2.27 [1.22 – 4.38]	0.011	2.10 [1.03 – 4.44]	0.046
Maximum SOMS level reached		0.13		0.40
2	1.36 [0.40 - 5.53]	0.64	1.65 [0.35 - 9.92]	0.55
3	2.26 [0.67 - 9.08]	0.21	2.09 [0.43 - 13.0]	0.39
4	3.49 [0.91 - 15.6]	0.08	3.47 [0.61 - 24.9]	0.18

Early mobilization is defined as mobilization within 72h of ICU admission.

OR Odds Ratio, *CI* Confidence interval, *SOMS* Surgical Intensive Care Unit Optimal Mobilization Score

a Model was corrected for postoperative care, frailty, department

b Model was corrected for Sepsis-related Organ Failure Assessment Score, body mass index (categories), postoperative, frailty, other admission reasons, admission, and non-traumatic brain injury

c Model was corrected for department and Glasgow Coma Scale

d Model was corrected for APACHE II, department, admission, Mobility-Transfer-Barthel Score at hospital admission and age (categories)

Additional File Table S6: Sensitivity Analysis with the ICU Mobility Scale.

	**Severely ill & Frail**		**Middle-aged**		**Young Trauma**		**Old non-frail**	
	**OR [95% CI]**	**p value**	**OR [95% CI]**	**p value**	**OR [95% CI]**	**p value**	**OR [95% CI]**	**p value**
Frequency of mobilization session per day	3.64 [0.96 - 15.7]	0.07	1.80 [0.91 - 3.66]	0.10	0.86 [0.20 - 3.60]	0.84	1.86 [0.93 - 3.82]	0.08
Mean duration per day (minutes)	1.03 [0.95 - 1.12]	0.45	1.00 [0.97 - 1.04]	0.84	0.98 [0.92 - 1.04]	0.54	1.04 [1.01 - 1.09]	0.025
Early mobilization	2.49 [0.92 - 7.11]	0.08	3.12 [1.77 - 5.61]	<0.001	6.68 [2.43 - 20.5]	<0.001	1.85 [1.00 - 3.55]	0.06
Maximum IMS level reached		0.008		<0.001		0.030		0.037
1	0.00 [NA - 4e36]	>0.99	1.36 [0.29 - 7.32]	0.70	45267791 [0.00 - 7e246]	>0.99	9.95 [1.23 - 213]	0.06
2	0.00 [0.00 - 3e76]	>0.99	0.00 [0.00 - 24209]	0.98	4.85 [0.00 - 7e73]	>0.99	3.93 [0.34 - 90.7]	0.29
3	10.3 [1.80 - 196]	0.031	3.58 [1.08 - 16.4]	0.06	81539612 [0.00 - NA]	>0.99	6.63 [1.24 - 123]	0.07
4	0.00 [NA - Inf]	>0.99	3.99 [0.42 - 32.5]	0.19	31236587 [0.00 - 2e262]	>0.99	15.2 [1.56 - 349]	0.031
5	18.9 [2.86 - 377]	0.009	14.7 [4.32 - 68.4]	<0.001	45418685 [0.00 - NA]	>0.99	12.2 [2.28 - 228]	0.018
6	71.8 [1.83 - 3881]	0.016	6.20 [1.01 - 41.8]	0.049	3e15 [0.00 - Inf]	>0.99	16.7 [2.18 - 353]	0.018
7	49.4 [3.35 - 1376]	0.006	38.1 [5.12 - 412]	<0.001	19150009 [0.00 - 2e236]	>0.99	10.6 [1.24 - 231]	0.053
8	0.00 [NA - Inf]	>0.99	13.0 [2.01 - 107]	0.010	2e15 [0.00 - NA]	0.99	49.8 [3.84 - 1452]	0.006
9	21.4 [1.64 - 558]	0.026	18.5 [5.00 - 91.9]	<0.001	55199593 [0.00 - NA]	>0.99	24.2 [3.84 - 478]	0.004
10	2.5e+10 [0.00 - NA]	>0.99	12.8 [2.13 - 93.6]	0.007	189210174 [0.00 - NA]	>0.99	10.0 [0.29 - 373]	0.17

Early mobilization is defined as mobilization within 72h of ICU admission.

OR Odds Ratio, CI Confidence interval. *IQR* Interquartile Range, *IMS* ICU Mobility Scale.

Additional File Table S7: Secondary Endpoints.

	Cluster					Post-hoc Analyses					
	Severely ill & Frail (N = 188)	Middle-aged (N = 331)	Young Trauma (N = 108)	Old non-frail (N = 321)	p value	Severely ill & Frail vs. Middle-aged	Severely ill & Frail vs. Young Trauma	Severely ill & Frail vs. Old non-frail	Middle-aged vs. Young Trauma	Middle-aged vs. Old non-frail	Young Trauma vs. Old non-frail
Mortality (ICU)	68 (36.2)	26 (7.9)	5 (4.6)	45 (14.0)	<0.001	<0.001	<0.001	<0.001	>0.9	0.07	0.051
Mortality (hospital)	89 (47.3)	38 (11.5)	5 (4.6)	71 (22.1)	<0.001	<0.001	<0.001	<0.001	0.2	0.002	<0.001
ICU length of stay (days)	15 [8 – 23]	10 [5 - 23]	10 [5 - 29]	11 [4 - 22]	0.005	0.007	0.3	0.005	>0.9	>0.9	>0.9
ICU length of stay (days) (survived patients)	15 [8 - 25]	9 [4 - 22]	11 [5 - 28]	9 [4 - 20]	0.013	0.025	>0.9	0.009	>0.9	>0.9	>0.9
Hospital length of stay (days)	27 [18 - 39]	29 [18 - 45]	26 [16 - 40]	28 [19 - 43]	0.37	>0.9	>0.9	>0.9	>0.9	>0.9	0.7
Hospital length of stay (days) (survived patients)	33 [22 - 48]	29 [18 - 45]	26 [16 - 42]	29 [20 - 44]	0.10	0.5	0.14	>0.9	>0.9	>0.9	0.4

Secondary endpoints in dependence of the four clusters. Here, the results of the primary analysis as well as the post-hoc analyses are listed.

Numbers are presented as n (%) or median [IQR]. *IQR* Interquartile Range, *ICU* Intensive Care Unit.
